# Investigation of Ionic Liquid interaction with ZnBDC-Metal Organic Framework through Scanning EXAFS and Inelastic Neutron Scattering

**DOI:** 10.1038/s41598-019-51344-0

**Published:** 2019-10-14

**Authors:** Rituraj Dutta, Mala N. Rao, Ashok Kumar

**Affiliations:** 1 Department of Physics, Tezpur University, Napaam, 784028 India; 2 Solid State Physics Division, Bhabha Atomic Research Centre, Mumbai, 400085 India; 3 Homi Bhabha National Institute, Mumbai, 400094 India

**Keywords:** Organic-inorganic nanostructures, Structure of solids and liquids

## Abstract

Zn based Metal-Organic Framework (MOF), Zinc 1,4-benzenedicarboxylate (ZnBDC) has been synthesized and incorporated with ionic liquid (IL) 1-butyl-3 methylimidazolium bromide (BMIMBr) at optimum 50 wt% of IL. Interaction of BMIMBr in the micropores of ZnBDC-MOF is investigated by XPS, scanning EXAFS and inelastic neutron spectroscopy. Significant increase in binding energies of Zn spin-orbit peaks upon IL incorporation is observed from XPS spectra indicating motion of Br^−^ anions of the IL BMIMBr towards the unsaturated Zn cluster of ZnBDC-MOF. The *k*-space periodicity as well as the local coordination geometry of Zn K-edge is investigated from the scanning EXAFS and XANES spectra. Asymmetric oscillation periodicity has been observed from the *k*-space scanning XANES spectra upon IL incorporation. Difference in peak positions of oxygen and zinc are observed from the R-space scanning EXAFS spectra suggesting change in coordination geometry due to dehydration of Zn^2+^ ion in the ZnBDC-MOF upon IL incorporation. Incorporation of IL in the pores of ZnBDC-MOF gives rise to increased scattering intensity in the Inelastic Neutron Scattering (INS) spectra, which is attributed to the displacement of IL ions in the MOF pores.

## Introduction

Metal-Organic Frameworks (MOFs) are hybrid microporous group of materials consisting of inorganic unsaturated metal nodes coordinated with organic linkers^1,2^. Owing to their large pore volume and adequate surface area MOFs are efficient nanomaterials for different sensing, catalytic, gas storage and sequestration applications^3^. Yafei He *et al*^4^. studied the development of porous carbon nanosheets (PCNs) from mesoporous layered materials including zeolites with controlled pore textures that show excellent electrochemical performances in energy-harvesting applications. MOFs are suitable host materials to entrap tiny guest molecules within their pores and to maintain their dynamics by tuneable interaction with the guest materials. Ionic Liquids (ILs) are molten salts with organic cations and inorganic anions having unlimited structural variations^5^. They have applications in various emerging fields owing to their special properties such asexcellent ionic conductivity (~10^−2^ S cm^−1^), low vapour pressure (~10^−10^ Pa at 300 K), wide electrochemical window (~6 V) and thermal stability (~300 °C), non-flammability and low toxicity^6^. ILs can be used as suitable guest materials to incorporate within the pores of MOFs. The tuneable interactions between ions of MOFs and ILs can maintain the phase dynamics and physicochemical properties of the nanocomposite by nano-sizing of guest ILs^7^. Yifei Chen *et al*. reported the experimental and theoretical work on Zn based MOF (IRMOF-1) incorporated with IL BMIMPF_6_ revealing the interaction of BMIM^+^ cations with the organic linker moieties and PF_6_^−^ anions with the unsaturated Zn metal nodes^8^.

X-ray Photoelectron Spectroscopy (XPS) is an efficient technique to determine the elemental composition of MOFs as well as the structural change in inorganic metal nodes upon incorporation of guest molecules. Wenbo Lu *et al*. reported the XPS measurements to investigate the elemental components of Ni based MOFs^9^. Yuxiu Sun *et al*. studied the grafting of ionic liquid into Cr based MOF by XPS for quick fixation of CO_2_^10^. Extended X-Ray absorption fine structure (EXAFS) and X-ray absorption near edge structure (XANES) are promising techniques to determine the coordination of the metal clusters of MOFs and their tuneable interaction with incorporated molecules. Quan Zuo *et al*^11^. investigated the Pt-N coordination through XPS, XANES and EXAFS and reported the ultrahigh loading of Pt in ultrathin nanosheets of Cu based MOF for efficient photocatalytic performance. C. Prestipino *et al*^12^. reported the Cu K-edged XANES and EXAFS of dehydrated HKUST-1 to investigate the local Cu^2+^ structure. Comparative studies on Zr K-edged XANES and EXAFS observed by Xinxin Sang *et al*^13^. to elucidate the rate of crystallization of UiO-66 MOFs dissolved separately in ionic liquid and N-N-dimethyl formamide (DMF). Luisa Sciortino *et al*^14^. reported the coordination structure of FeBTC-MOF by EXAFS and XANES and confirmed that the unsaturated metal building units have Fe^3+^ states. F. L. Morel *et al*^15^. reported the XANES spectra at P K-edge of phosphine and phosphine oxide functionalized framework materials to investigate their structural topology. M. Muller *et al*^16^. studied the EXAFS and XANES on Zn based MOF after incorporation of ZnO and Cu based nanoparticles to be used as effective catalyst for facile synthesis of methanol. Xinzuo Fang *et al*^17^. investigated the local coordination and stabilization of Pt atoms in pores of Al based MOF through EXAFS analysis and reported the confinement of single Pt atoms in the MOF.

To investigate the phase behaviour as well as the ionic transport in the composite, the role of MOF-IL interaction is utmost necessary. Neutron Inelastic Scattering (INS) spectroscopy can reveal the probable interaction of IL ions with the MOF. Also the modulation or shifting of defect sites present in the MOF can also be investigated to enhance the microporosity for better entrapping of ions of ILs. Neutron scattering spectra can determine any structural defect or shifting of any functional group present in the metal clusters or in the organic linkers of MOF. Hui Wu *et al*^18^. studied the role of acetic acid as modulator in UiO-66 (Zr-MOF) through Neutron inelastic scattering. S. Yang *et al*^19^. studied the neutron scattering spectra of MFM-300 (Al) loaded by H_2_ to investigate the rotational recoil motion of adsorbed H_2_ in the pores. Y. Liu *et al*^20^. reported the experimental neutron scattering analysis of D_2_ and para hydrogen in MOF-74-Fe to elucidate the probability of pair formation. Due to excess incoherent scattering cross section of H_2_, the dynamics of H_2_ dominates the inelastic neutron scattering (INS) spectra. Rosi *et al*^21^. and Rowsell *et al*^22^. attempted to identify the hydrogen binding sites in ZnBDC-MOF through INS. Spencer *et al*^23^. employed single crystal neutron diffraction to elucidate the adsorption characteristics of H_2_ in MOF-5. Two primary adsorption sites were located and one site was in agreement with that derived from INS data. First-principle lattice dynamics computations and INS measurements of the density of states of phonons have been reported by Zhou and Yildirim^24^. The origin of the negative thermal expansion in ZnBDC-MOF has been investigated by Lock *et al*^25^. through x-ray as well as neutron diffraction and INS spectroscopy.

Studies on different guest or substituted IL incorporation to MOFs have been reported^26^, but no study on INS spectroscopy of IL incorporated MOF has been reported yet. In this work, ZnBDC-MOF (MOF-5) has been incorporated with IL 1-butyl-3 methylimidazolium bromide at optimum 50 wt% of IL with a view to study the confinement of ions of BMIMBr in the pores of ZnBDC-MOF. The interaction of IL ions with the Zn metal nodes or the linker moieties of ZnBDC-MOF has been analyzed through Scanning EXAFS, XPS and Inelastic Neutron Scattering spectroscopy.

## Results

### X-ray diffraction analysis

**Figure 1:**
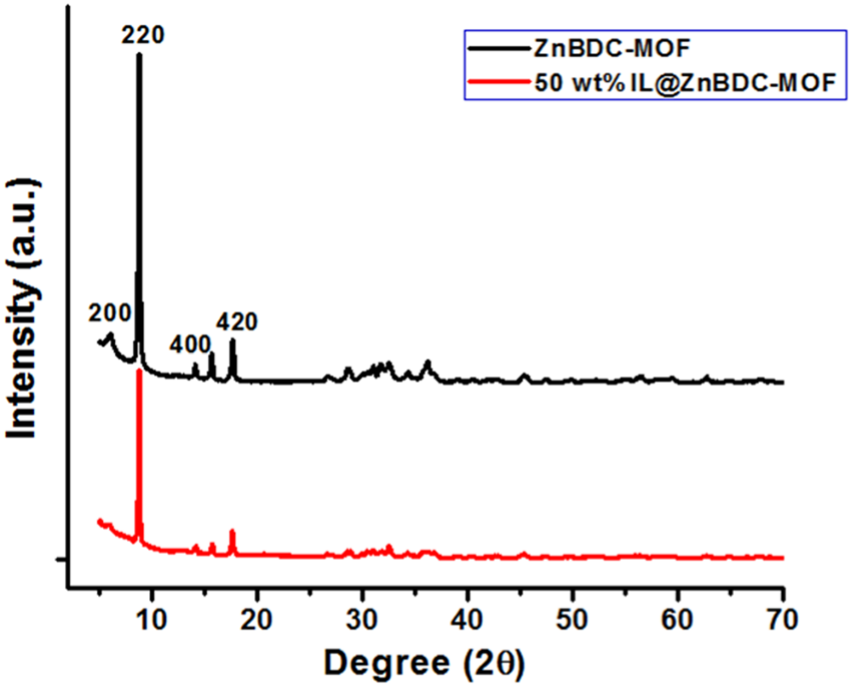
X-Ray Diffraction patterns of ZnBDC-MOF and 50 wt% IL incorporated ZnBDC-MOF nanocomposite.

The XRD patterns of ZnBDC-MOF and 50 wt% of IL incorporated ZnBDC-MOF nanocomposites are shown in Fig. 1. The characteristic peaks at 2θ = 6° correspond to the (200) planes, 2θ = 9° correspond to the (220) planes, 2θ = 13.7° correspond to the (400) planes and 2θ = 17.8° correspond to (420) planes of pristine ZnBDC-MOF^27^.

The intensities of the crystalline peaks decrease with incorporation of IL due to the enhancement in amorphicity of the composite system. Using Scherrer equation, the crystallite size (L) of the nanocomposite has been calculated as,

1 $${\rm{L}}={\rm{K}}\lambda /\beta \,\cos \,\theta $$

where, K is the constant of crystallinity with value 0.89, λ is the corresponding wavelength of X-Ray (1.54 Å), *β* is the full width at half maximum (FWHM) of any diffraction peak at angle 2θ. With incorporation of IL in ZnBDC-MOF, the FWHM increases to 0.17 from 0.13 as for pristine ZnBDC-MOF. Consequently the crystallite size of the nanocomposite decreases to 8.11 Å from 10.62 Å as for pristine ZnBDC-MOF.

### FTIR Analysis

**Figure 2:**
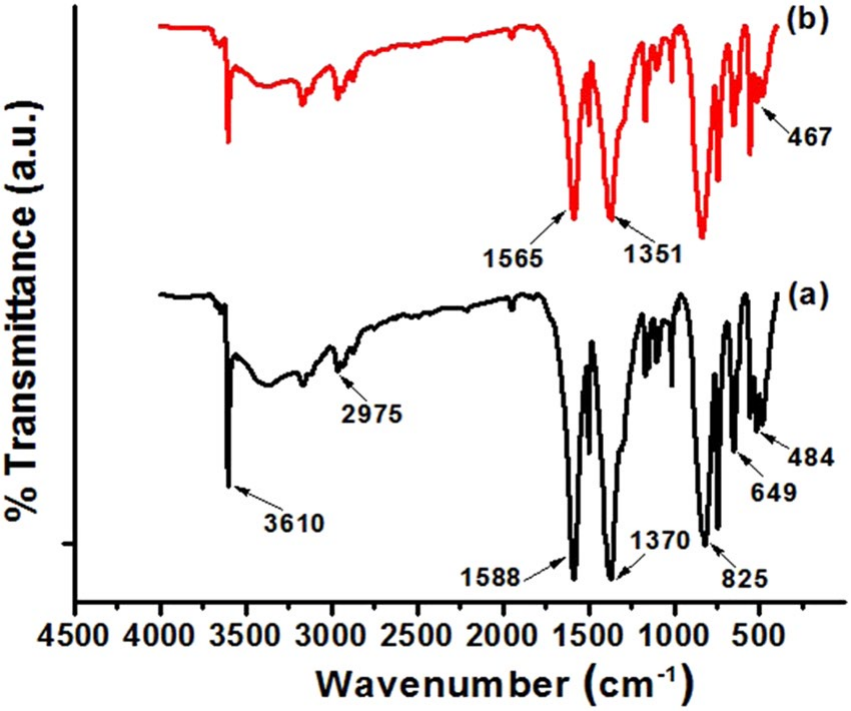
FTIR Spectra of (**a**) ZnBDC-MOF and (**b**) 50% IL incorporated ZnBDC-MOF nanocomposite.

The FTIR spectra of pristine ZnBDC-MOF and 50 wt% of IL incorporated ZnBDC-MOF nanocomposites are depicted in Fig. 2. Due to the incorporation of IL into the pores of ZnBDC-MOF, the interaction between the Zn metal node and the organic linker moieties becomes weaker. The peaks corresponding to the symmetric and asymmetric vibrations of carboxyl groups (-COOH) present in the organic ligands of ZnBDC-MOF appear at 1370 cm^−1^ and 1588 cm^−1^ in the pristine ZnBDC-MOF before incorporation of IL. Due to the confinement of IL in the pores of ZnBDC-MOF, the peaks of carboxylate modes are shifting towards lower wavenumber positions upon incorporation of IL in the MOF. The peak at 1370 cm^−1^ shifts to 1351 cm^−1^ and the peak 1588 cm^−1^ shifts to 1565 cm^−1^ in the 50 wt% of IL incorporated ZnBDC-MOF nanocomposite^28^. Also the stretching vibrational mode of Zn-O shifts from 484 cm^−1^ to 467 cm^−1^ upon incorporation of IL.

One sharp peak at around 3610 cm^−1^ appears indicating the hydroxyl group (-OH) in ZnBDC-MOF^29^. The peak corresponding to out of plane C-H bending vibration of ZnBDC-MOF as well as imidazole ring of BMIMBr occurs at 825 cm^−1^ ^30^. At 649 cm^−1^, the peak represents the C-H bending vibration of aromatic linker in ZnBDC-MOF^31^.

### Morphological analysis

**Figure 3:**
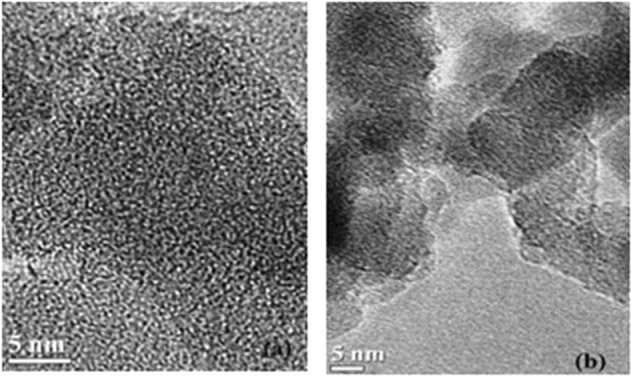
HRTEM Micrograph of (**a**) ZnBDC-MOF (**b**) 50 wt% IL incorporated ZnBDC-MOF.

In Fig. 3(a) ZnBDC-MOF nanoparticles with average size of 1.5 nm are observed. Incorporation of IL BMIMBr into the ZnBDC-MOF can fill up the empty pores. Interactions of Br^−^ anions with the Zn metal cluster and the BMIM^+^ cations with organic moieties of MOF reduce the pore spaces in the composite system. Some granular agglomerated particles are also present and that may form mesopores^7^.

### Thermal analysis

**Figure 4:**
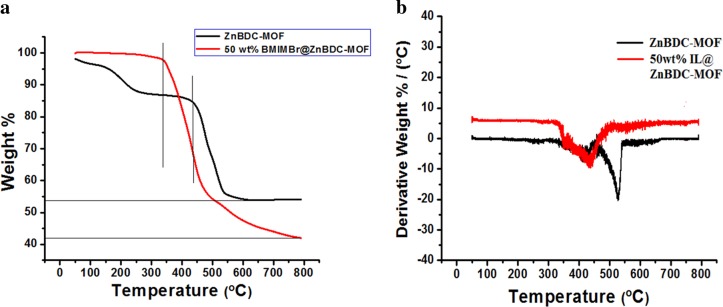
(**a**) Thermogravimetric analysis of pristine and IL incorporated ZnBDC-MOF nanocomposites. (**b**) Derivative of TG curves of pristine and IL incorporated ZnBDC-MOF nanocomposites.

Thermal stability analysis of pristine and 50 wt% IL incorporated ZnBDC-MOF is represented by Thermogravimetric plots in Fig. 4(a). The thermal stability decreases as the amorphicity of the nanocomposite increases with IL incorporation in the ZnBDC-MOF. The pristine ZnBDC-MOF shows thermal degradation at 440.4 °C while in case of 50% IL incorporated sample, thermal stability is degrading upto 339.6 °C. Also, weight loss (%) increases from 46.4 to 55.8 °C at 50 wt% IL incorporation in ZnBDC-MOF. Derivative of TG curves of pristine and IL incorporated ZnBDC-MOF nanocomposites are shown in Fig. 4(b). Reduction in onset as well as rapidest decomposition temperature is observed at 50 wt% IL incorporated ZnBDC-MOF nanocomposite as incorporation of IL can enhance the amorphicity of the nanocomposite system. The onset temperature decreases from 437 to 311 °C and the rapidest temperature decreases from 526 to 432 °C upon 50 wt% IL incorporation.

### N_2_ adsorption-desorption analysis

**Figure 5:**
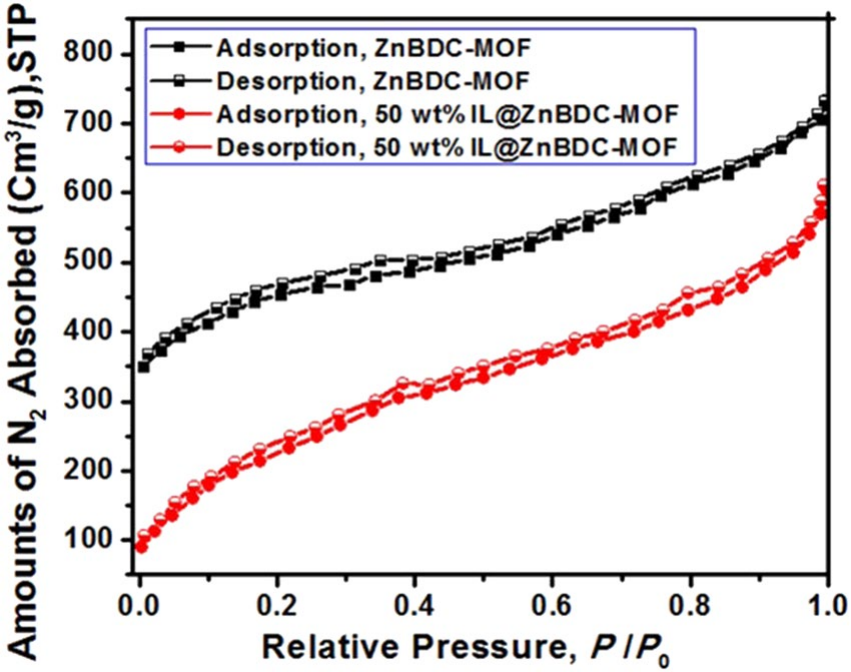
N_2_ adsorption-desorption isotherms of pristine and 50 wt% IL incorporated ZnBDC-MOF nanocomposite.

The N_2_ adsorption-desorption isotherms of pristine and 50 wt% IL incorporated ZnBDC-MOF nanocomposite are shown in Fig. 5. At 50 wt% of IL incorporation, N_2_ adsorption decreases at low relative pressure as ions of IL are mostly confined at micropores of ZnBDC-MOF rather than at mesopores^7^. MOFs have preferentially microporous structure of less than 2 nm and agglomeration of tiny micropores may form mesopores of 2 to 50 nm. Due to the agglomeration of ZnBDC-MOF as observed from the TEM results, granular sites may form within the porous structure. N_2_ adsorption at the agglomerated granular sites occurs because of the formation of mesopores. The pore volume of the nanocomposite has been determined by the Horvath- Kawazoe (HK) approximation which deals with the statistical evaluation of gas adsorption in different slit pores. According to this approximation, adsorption at pores occurs only at specific relative pressure depending on the interaction energy in between the adsorptive molecules and the adsorbent^32^. The pore volume (*V*) is calculated from the total surface area (*S*_*BET*_), empirical distribution of pore width (*D*) at various relative pressure of adsorption of N_2_ (77 K) as,

2 $$V=D.{S}_{BET}/4$$

The surface area of the nanocomposite decreases from 1071 m^2^/g to 750 m^2^/g and pore volume reduces from 0.40 cm^3^/g to 0.28 cm^3^/g upon incorporation of 50 wt% of IL in ZnBDC-MOF.

### X-ray photoelectron spectroscopy analysis

**Figure 6:**
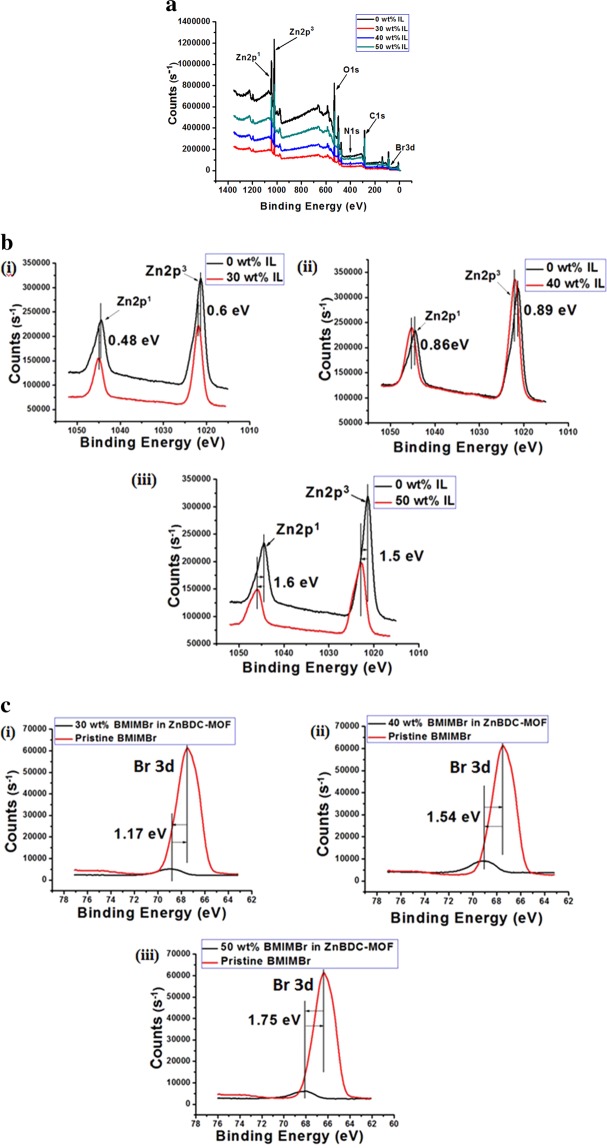
(**a**) The X-ray photoelectron spectroscopy spectra of ZnBDC-MOF nanocomposite at different wt% of BMIMBr. (**b**) X-ray photoelectron spectroscopy spectra of Zn 2p peaks for pristine ZnBDC-MOF (0 wt% IL) with (i) 30 wt% of IL incorporated ZnBDC-MOF nanocomposite, (ii) 40 wt% of IL incorporated ZnBDC-MOF nanocomposite and (iii) 50 wt% of IL incorporated ZnBDC-MOF nanocomposite. (**c**) X-ray photoelectron spectroscopy spectra of Br 3d peak for pristine BMIMBr with (i) 30 wt% of BMIMBr incorporated ZnBDC-MOF nanocomposite, (ii) 40 wt% of BMIMBr incorporated ZnBDC-MOF nanocomposite and (iii) 50 wt% of BMIMBr incorporated ZnBDC-MOF nanocomposite.

The X-ray Photoelectron Spectroscopy (XPS) spectra of pristine, 30 wt%, 40 wt% and 50 wt% IL incorporated ZnBDC-MOF nanocomposites have been depicted in Fig. 6(a). XPS spectra indicate the presence of Zn, C and O in pristine and the presence of Br and N in addition to Zn, C and O in the IL incorporated ZnBDC-MOF nanocomposites. The spin-orbit peaks corresponding to Zn 2p^1/2^ and Zn 2p^3/2^ appear at 1046.1 and 1026.2 eV, respectively in pristine ZnBDC-MOF that are observed to shift to higher binding energy positions with increasing IL incorporation as depicted in Fig. 6(b). Shift of 0.48 eV and 0.6 eV for 30 wt% IL incorporation, 0.86 eV and 0.89 eV for 40 wt% IL incorporation and 1.6 eV and 1.5 eV for 50 wt% IL incorporation in ZnBDC-MOF towards higher binding energies have been observed respectively for the Zn 2p^1/2^ and Zn 2p^3/2^peaks. The peak of O1s appears at 531.6 eV indicating unsaturated Zn-O metal cluster, while C 1 s peak appears at 287.4 eV indicating the benzene-1,4 dicarboxylic acid linker. The N 1 s peak appears at 400.5 eV and Br 3d peak appears at 70.9 eV in the XPS spectra.

Differences in binding energies of Br 3d peaks are observed to be 1.17 eV, 1.54 eV and 1.75 eV, respectively upon incorporation of 30 wt%, 40 wt% and 50 wt% IL in ZnBDC-MOF as compared to pristine 1-butyl-3-methylimidazolium bromide as depicted in Fig. 6(c).

### Scanning EXAFS and XANES analysis

Scanning EXAFS and XANES measurements have been carried out to investigate the absorption characteristics and coordination geometry of the Zn K-edge of ZnBDC-MOF upon incorporation of IL. In scanning EXAFS, the absorption coefficient *μ(E)* of any experimental sample in transmission mode is expressed as,

3 $$I={I}_{0}\,\exp \,[-\mu (E)x]$$

where, *I* is the transmitted photon beam from the surface, *I*_0_ is the incident photon beam and *x* is the distance travelled by the beam. The scanning EXAFS spectra were carried out in the energy range from 9600 to 10800 eV and scanning XANES spectra were from 9700 to 10000 eV. The absorption spectrum of reference zinc metal foil has been recorded prior to the experimental materials for the angle and energy calibrations.

**Figure 7:**
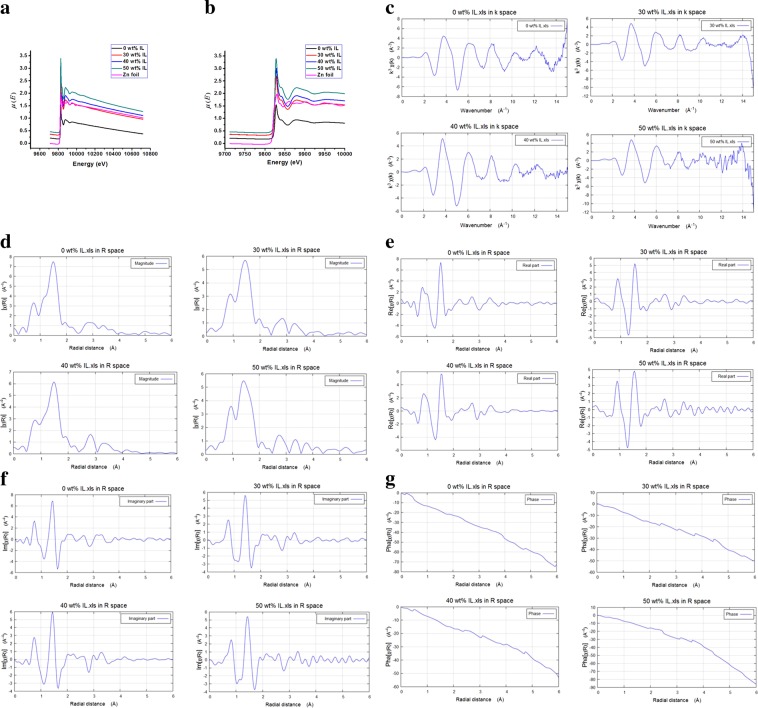
(**a**) Scanning EXAFS spectra of ZnBDC-MOF nanocomposite at different wt% of BMIMBr. (**b**) Scanning XANES spectra of ZnBDC-MOF nanocomposite at different wt% of BMIMBr. (**c**) *k*-space XANES spectra of ZnBDC-MOF nanocomposite at different wt% of BMIMBr. (**d**) Magnitude of R-space EXAFS spectra of ZnBDC-MOF nanocomposites at different wt% of BMIMBr. (**e**) Real part of R-space EXAFS spectra of ZnBDC-MOF nanocomposite at different wt5 of BMIMBr. (**f**) Imaginary part of R-space EXAFS spectra of ZnBDC-MOF nanocomposite at different wt% of BMIMBr. (**g**) Phase of R-space EXAFS spectra of ZnBDC-MOF nanocomposite at different wt% of BMIMBr.

From the Scanning EXAFS spectra in Fig. 7(a), Zn K-edge peak of ZnBDC-MOF has been observed at 9827.7 eV that confirms the +2 oxidation state of Zn species. With increasing incorporation of IL from 30 wt% to 40 wt%, the oxidation state of Zn does not change as the K-edge peak positions remain unchanged. The absorption coefficient *μ*(*E*) of Zn K-edge increases from 1.52 to 3.42 at maximum 50 wt% IL incorporation indicating change in coordination geometry of Zn (II) species. The XANES data of ZnO (Zn foil) have been provided at Fig. 7(b) with pristine ZnBDC-MOF and 30 wt%, 40 wt% and 50 wt% IL incorporated ZnBDC-MOF nanocomposites. Zn K-edge peak of ZnO observed at 9827.7 eV confirms the +2 oxidation state of Zn. The K-edge peaks in the XANES spectra of pristine ZnBDC-MOF and 30 wt%, 40 wt% and 50 wt% IL incorporated ZnBDC-MOF are also observed at 9827.7 eV indicating that Zn species in pristine ZnBDC-MOF as well as upon IL incorporation remain in +2 oxidation state. In Scanning XANES spectra in Fig. 7(b) the first resonance peak after the Zn K-edge is clearly observed around 9878.6 eV, which is attributed to Zn-O bonding. To investigate the oscillation periodicity as well as coordination of Zn metal node upon increasing wt% of IL incorporation, the *k*-space XANES in Fig. 7(c) and magnitude of R-space EXAFS in Fig. 7(d) have been obtained using Athena software. With increasing wt% of IL incorporation from 30 wt% to 50 wt%, the *k*-space peak amplitudes are observed to change with occurrence of heterogeneity or mismatch in periodicity. In magnitude of R-space, two distinct peaks at 0.95 and 1.61 Å are observed with respect to oxygen and zinc in pristine ZnBDC-MOF that shift to 0.86 and 1.55 Å upon maximum 50 wt% of IL incorporation^13^. To understand the changes in coordination geometry of Zn^2+^ ion in the ZnBDC-MOF upon different wt% of IL incorporation, the real and imaginary parts of R-space EXAFS data along with the R-space phase behaviour have been provided in Fig. 7(e–g) for pristine ZnBDC-MOF and 30 wt%, 40 wt% and 50 wt% IL incorporated ZnBDC-MOF nanocomposites. In real and imaginary parts of R-space EXAFS data, asymmetry in Fourier transform peak amplitudes has been observed upon increasing IL incorporation as compared to pristine ZnBDC-MOF along the radial distance from 0 to 6 Å. R-space phase dynamics also changes upon increasing IL incorporation from 30 wt% to 50 wt% in ZnBDC-MOF. Moreover, mismatch or heterogeneity is observed in Fourier transform data corresponding to radial distance with increasing wt% of IL incorporation in ZnBDC-MOF nanocomposites as compared to the pristine one.

### Inelastic neutron scattering analysis

INS measurement reveals the dynamical processes in materials and the measured scattering intensity is directly related to the displacement of the scattering atom. In solid state ion conducting materials, the ion migration is enhanced by phonon assisted diffusion. The inelastic neutron scattering can probe the low energy phonon modes and the energy range (below 50 meV) is highly accessible that cannot be investigated by Infrared and Raman Spectroscopy techniques^33^. At low energy lattice excitation, the displacement of the diffusing ion occurs according to the polarization affect of the involved specific phonon resulting in a strongly anisotropic diffusion path. The mobile ions follow different paths within the lattice as a function of ion content and temperature resulting in long-range complex structures or disordered short-range arrangements^34^.

In INS experiment, the differential cross section is related to the dynamical structure factor or correlation function *S*(*Q, ω*) as,

4 $$\frac{{d}^{2}\sigma }{d\Omega dE}=N\frac{{k}_{f}}{{k}_{i}}S(Q,\omega )$$

where,

5 $$S(Q,\omega )=\frac{1}{2\pi \hslash N}{\sum }_{{u}^{/}}{\int }_{-\propto }^{\propto }{\rm{dt}}\langle {e}^{-iQ.{r}_{1}^{/}(0)}{e}^{iQ.{r}_{1}(t)}\rangle {e}^{-i\omega t}$$

*N* is the total number of nuclei, $\frac{{k}_{f}}{{k}_{i}}$ is the experimental set up dependent term, the term $\langle {e}^{-iQ.{r}_{1}^{/}(0)}{e}^{iQ.{r}_{1}(t)}\rangle$ represents the correlation of positions of two nuclei of which one is at time *t* = 0 and other one at a later time *t*^35^.

**Figure 8:**
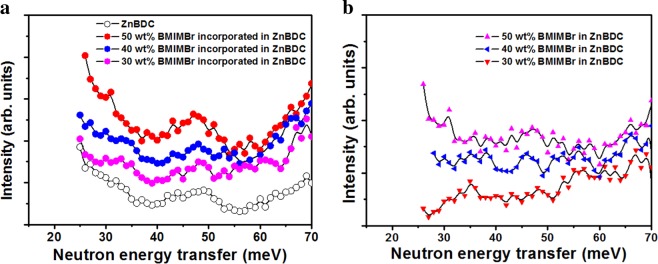
(**a**) INS spectra of ZnBDC-MOF and 30 wt%, 40 wt% and 50 wt% BMIMBr incorporated in ZnBDC-MOF. (**b**) Difference spectrum for 30 wt%, 40 wt% and 50 wt% BMIMBr in ZnBDC-MOF.

The powder samples of pristine ZnBDC-MOF and 30 wt%, 40 wt% and 50 wt% of IL BMIMBr incorporated ZnBDC were kept in an aluminium container during the measurements. The INS spectrum of the reference MOF sample was collected, followed by data acquisition with the MOF loaded with 30 wt%, 40 wt% and 50 wt% of ionic liquid. Assignments of the spectra of the ionic liquid were carried out by the subtraction of the spectrum collected from the pristine ZnBDC-MOF sample. The INS spectra cover a large energy range (upto 180 meV), in accordance with results in Zhou and Yildirim^24^, but data up to 70 meV are shown in Fig. 8(a),(b). Lock *et al*^25^. determined three spectral lines at approximately 46, 50 and 57 meV for ZnBDC. Signatures of these lines are seen in Fig. 8(a). The phonon modes that contribute in this energy range are of twisting, stretching, breathing, bending, and wagging motions including the translational in-phase transverse (trampoline) motion and out-of-phase rotational motion of the aromatic ring of the benzene rings of the BDC linker molecules of the MOF^25^. The INS spectra for 50 wt% IL incorporated ZnBDC-MOF depict increased scattering intensities compared to that for pristine ZnBDC-MOF, 30 wt% and 40 wt% IL incorporated ZnBDC-MOF nanocomposites. The difference spectra for 30 wt%, 40 wt% and 50 wt% BMIMBr in ZnBDC-MOF are shown in Fig. 8(b).

## Discussion

Amorphicity of the nanocomposite increases in XRD patterns after IL incorporation due to the interaction of BMIM^+^ cations of IL with the linker moieties of MOF and Br^−^ anions of IL with the unsaturated Zn metal cluster of MOF. The shifting of carboxylate modes in FTIR spectra can be attributed to the bond elongation between the inorganic metal cluster and the organic linker moieties due to interaction of Br^−^ anions of IL with the Zn metal nodes. In BET analysis, upon IL incorporation the cations BMIM^+^ tend to occupy the open pore spaces of MOF resulting in reduction in total surface area as well as the effective pore width of the nanocomposite system. Shifting of Zn spin-orbit peaks towards higher binding energy positions in XPS spectra with increasing wt% of IL is due to the electrostatic interaction of Zn metal nodes with Br^−^ anions of IL because of which the anions tend to shift towards the Zn node causing increase in the binding energies. The binding energy difference in the Zn metal node upon increasing wt% of IL incorporation indicates the affinity of Br^−^ anions of IL towards the Zn metal node in the pores of ZnBDC-MOF. Shifting of Br peaks in XPS spectra is also observed with increasing wt% of IL from 30 wt% to 50 wt% as compared to that in the pristine IL 1-butyl-3-methylimidazolium bromide, which is attributed to the interaction of Br^−^ anions with Zn metal nodes of the ZnBDC-MOF. Heterogeneity in periodic oscillation in *k*-space scanning XANES data is attributed to degradation of first shell Zn-O bonding due to dominant electrostatic interaction of unsaturated Zn metal nodes with Br^−^ anion with increasing wt% of IL in ZnBDC-MOF. Shifting of peaks of oxygen and zinc in R-space scanning EXAFS data indicates the dehydration of Zn metal ion with increasing wt% of IL incorporation that in turn changes the coordination geometry. Heterogeneity or mismatch observed in R-space Fourier transform peak amplitudes and phase upon increasing wt% of IL incorporation indicates the changes in coordination geometry of Zn^2+^ ions in ZnBDC-MOF. This can be attributed to the deformation of coordination structure of Zn metal nodes upon increasing wt% of IL incorporation due to the weakening of Zn-O bonding by the dominant interaction of Br^−^ anions of IL with Zn^2+^ cations in ZnBDC-MOF. Weakening of Zn-O bonding giving rise to Zn-O bond elongation after incorporation of IL is also confirmed by shifting of the stretching vibrational mode of Zn-O from 484 cm^−1^ to lower wavenumber of 467 cm^−1^ in the FTIR results. In INS spectra though the spectral features are not well differentiated, the presence of 50 wt% of IL in the ZnBDC-MOF gives rise to increased scattering intensities as compared to that of the pristine ZnBDC-MOF, 30 wt% and 40 wt% IL incorporated ZnBDC-MOF nanocomposites. The scattering intensity of the peaks corresponding to the spectral lines at 46, 50 and 57 meV are observed to increase with increasing concentration of IL from 30 wt% to 50 wt% indicating the displacement of ions of IL in the pores of ZnBDC-MOF. In the difference spectra of INS [Fig. 8(b)], the scattering intensity of the peaks corresponding to 35.2, 55.8 and 66.2 meV are observed to increase with increasing IL incorporation from 30 wt% to 50 wt% in the ZnBDC-MOF suggesting the interaction of ions of IL with MOF. At 10 wt% and 20 wt% of IL incorporation in ZnBDC-MOF, INS signals with weak scattering intensity are observed and not much difference is obtained from the difference spectra of INS as compared to pristine ZnBDC-MOF. Below 30 wt% of IL incorporation in ZnBDC-MOF, the less number of pores are filled resulting in weak scattering intensity profiles in INS spectra. Beyond 50 wt% of IL incorporation in the ZnBDC-MOF, the composite becomes viscous due to excess IL loading outside the pores as described in the synthesis procedure of the nanocomposites.

## Methods

### Materials

Zinc Nitrate Hexahydrate was procured from Avantor Performance Materials Ltd., 1,4-Benzene dicarboxylic acid and 1-butyl 3-methylimidazolium bromide were purchased from Sigma Aldrich. N-N-Dimethyl Formamide, Methanol and Triethylamine were purchased from Merck Specialities Pvt. Ltd. No further purification was done to the materials before use in the synthesis process.

### Synthesis of ZnBDC-MOF

ZnBDC-MOF has been synthesized by solvothermal synthesis method. Zinc Nitrate Hexahydrate (1.35 g) and 1,4-benzene dicarboxylic acid (0.25 g) were dissolved in 150 ml of N, N^/^-dimethyl formamide (DMF). After room temperature stirring for 30 minutes, 1 ml of triethylamine (TEA) was injected into the precursor^26^. After 24 hrs stirring the solution was washed 3 times with DMF and methanol to decant the unreacted part of Zinc Nitrate. Finally the precipitate was dried for 12 hrs under vacuum at 150 °C and 1.3 g crystalline yield was obtained.

### Incorporation of IL in ZnBDC-MOF

30 wt%, 40 wt% and optimum 50 wt% of IL BMIMBr has been incorporated into ZnBDC-MOF by direct mixing using mortar and pestle. Beyond 50 wt% of IL incorporation in the ZnBDC-MOF, the composite becomes viscous due to excess IL loading in the MOF, which may not be accommodated within the pores of MOF and remains outside making the composite viscous. For homogeneous distribution of ions of IL into the micropores of ZnBDC-MOF, the nanocomposite was vacuum heated at 100 °C for 12 hrs.

### Characterization techniques

Powder XRD patterns of ZnBDC-MOF and IL incorporated ZnBDC-MOF nanocomposites were recorded by Bruker AXS D8 X-ray Diffractometer at the scan rate of 1°/min and in the 2*θ* range of 5° to 70°. The FTIR spectra were recorded by Nicolet Impact 410 FTIR spectrometer in 400 to 4500 cm^−1^ wave number range. The morphology the nanocomposites were investigated by the High Resolution Transmission Electron Microscope (HRTEM) (Model: TECNAI G2 20 S-TWIN). The N_2_ adsorption-desorption isotherms of the samples were obtained by the BET Surface area analyzer (NOVA 1000E). The thermogravimetric measurements were carried out by STA 6000 Thermal Analyzer.

The scanning XANES and EXAFS data were collected in transmission mode by monochromatic synchrotron beamline with a source of 2.5 GeV bending magnet operating with flux of 10^11^ photon/sec at Indus-2 at Raja Ramanna Centre for Advanced Technology, (RRCAT) Indore, India. The incident photon of the synchrotron beamline has the energy resolution (*E* /∆*E*) of 10^4^ with a beam size of 1 mm × 0.2 mm. The elemental compositions as well as binding energy of the nanocomposites were investigated by ESCALAB Xi+ XPS instrument at CSIR-NEIST, Jorhat, India. The Inelastic Neutron Scattering experiment was carried out on Triple Axis Spectrometer in the energy loss mode with constant momentum transfer (Q) at Dhruva reactor, Bhabha Atomic Research Centre (BARC), Mumbai, India. A monochromatic neutron beam is incident on the sample and the neutrons with energies within a small band (0–70 meV) determined by diffraction from a graphite analyzer reach the detector.

## Conclusion

Confinement of IL ions in the micropores of ZnBDC-MOF has been investigated with incorporation of optimum 50 wt% of BMIMBr. XRD patterns reveal the reduction in intensity as well as crystallite size upon IL incorporation in the nanocomposite. FTIR spectra show the stretching and bending vibrations of different functional groups present in the nanocomposite system and shifting of −COOH symmetric and asymmetric vibrational bands towards lower wave numbers is observed due to interaction of Br^−^ ions of IL with the unsaturated metal nodes of ZnBDC-MOF. HRTEM micrographs depict compact structures of the nanocomposite due to decrease in pore spaces by the incorporation of ionic liquid. Reduction in thermal stability of the nanocomposite upto 339.6°C is observed from TGA due to enhanced amorphicity of the nanocomposite upon IL incorporation. BET analysis depicts the reduction in surface area as well as pore volume of the nanocomposite upto 750 m^2^/g and 0.28 cm^3^/g respectively, at 50 wt% IL incorporation as the pores of the ZnBDC-MOF are filled by the ions of IL. The XPS spectra reveal the shifting of Zn spin-orbit peaks towards higher binding energy sites with increasing IL incorporation from 30 wt% to 50 wt% as the Br^−^ anions of IL tend to move towards Zn metal nodes. Shifting of Br peaks is also observed in the XPS spectra with increasing wt% of IL upto 50 wt% as compared to that in the pristine IL, which is due to the interaction of Br^−^ anions with Zn metal nodes of the ZnBDC-MOF. From scanning XANES and EXAFS results, asymmetry in *k*-space periodicity and R-space phase with change in coordination geometry has been observed at Zn K-edge with increasing IL incorporation from 30 wt% to 50 wt%, which is attributed to displacement of Br^−^ anions towards the Zn metal nodes of ZnBDC-MOF. Incorporation of 50 wt% of IL BMIMBr in ZnBDC-MOF shows increased scattering intensity in the INS spectra as compared to that for pristine ZnBDC-MOF as well as 30 wt% and 40 wt% of IL incorporated ZnBDC-MOF nanocomposites indicating the displacement of IL ions triggered the MOF lattice excitations.
